# Cancer Associated Fibroblast (CAF) Regulation of PDAC Parenchymal (CPC) and CSC Phenotypes Is Modulated by ECM Composition

**DOI:** 10.3390/cancers14153737

**Published:** 2022-07-31

**Authors:** Stefania Cannone, Maria Raffaella Greco, Tiago M. A. Carvalho, Helene Guizouarn, Olivier Soriani, Daria Di Molfetta, Richard Tomasini, Katrine Zeeberg, Stephan Joel Reshkin, Rosa Angela Cardone

**Affiliations:** 1 Department of Biosciences, Biotechnology and Biopharmaceutics, University of Bari, 70126 Bari, Italy; stefaniacannone92@gmail.com (S.C.); grecoraffaella1975@gmail.com (M.R.G.); tiago.amaralcarvalho@uniba.it (T.M.A.C.); daria.dimolfetta@uniba.it (D.D.M.); katrinezeeberg@gmail.com (K.Z.); 2 Institute of Biology de Valrose, CNRS UMR 7277, University of Nice, 06108 Nice, France; helene.guizouarn@univ-cotedazur.fr (H.G.); olivier.soriani@unice.fr (O.S.); 3 INSERM, U1068, Centre de Recherche en Cancérologie de Marseille, Institut Paoli-Calmettes, CNRS, UMR7258, 13009 Marseille, France; richard.tomasini@inserm.fr

**Keywords:** desmoplastic reaction, pancreatic ductal adenocarcinoma, vasculogenic mimicry, 3D organotypic cultures, invadopodia

## Abstract

**Simple Summary:**

Here, we demonstrate for the first time that ECM composition cooperates with CAFs to jointly regulate/modulate the highly dynamic interactions between the CPC and CSC cell lines and establish a continuum between tumor initiation and progression in primary PDAC tumors. Altogether, these findings propose a scenario in which the ECM composition and the cellular secretome of the CAFs cooperate to jointly regulate both growth and morphology of the CPC and CSC cell lines and, by modulating the highly dynamic interactions between them, establishes a continuum between tumor initiation and progression in primary PDAC tumors.

**Abstract:**

Background: Pancreatic ductal adenocarcinoma (PDAC) is one of the deadliest of all cancers, having one of the lowest five-year survival rates. One of its hallmarks is a dense desmoplastic stroma consisting in the abnormal accumulation of extracellular matrix (ECM) components, especially Collagen I. This highly fibrotic stroma embeds the bulk cancer (parenchymal) cells (CPCs), cancer stem cells (CSCs) and the main producers of the stromal reaction, the Cancer Associated Fibroblasts (CAFs). Little is known about the role of the acellular ECM in the interplay of the CAFs with the different tumor cell types in determining their phenotypic plasticity and eventual cell fate. Methods: Here, we analyzed the role of ECM collagen I in modulating the effect of CAF-derived signals by incubating PDAC CPCs and CSCs grown on ECM mimicking early (low collagen I levels) and late (high collagen I levels) stage PDAC stroma with conditioned medium from primary cultured CAFs derived from patients with PDAC in a previously described three-dimensional (3D) organotypic model of PDAC. Results: We found that CAFs (1) reduced CPC growth while favoring CSC growth independently of the ECM; (2) increased the invasive capacity of only CPCs on the ECM mimicking the early tumor; and (3) favored vasculogenic mimicry (VM) especially of the CSCs on the ECM mimicking an early tumor. Conclusions: We conclude that the CAFs and acellular stromal components interact to modulate the tumor behaviors of the PDAC CPC and CSC cell types and drive metastatic progression by stimulating the phenotypic characteristics of each tumor cell type that contribute to metastasis.

## 1. Introduction

Pancreatic Ductal Adenocarcinoma (PDAC) is one of the most lethal cancers, having a five-year survival rate of less than 8% [1,2], and will become the second most common cause of cancer deaths in the coming years [3,4].

One of the aggressive hallmarks of PDACs is their prominent, highly reactive stromal microenvironment named desmoplasia, which makes up to 90% of PDAC tissue and supports tumor progression [5,6,7,8,9,10]. Desmoplasia is a dense extracellular matrix (ECM) with ever more collagen type I as the tumor progresses and in which are embedded the cancer cells and their accessory cells, including cancer associated fibroblasts (CAFs) [11,12]. In PDAC, collagen I can constitute up to 80% of the tumor space, is associated with a worsened outcome [13,14] and stimulates malignant cell properties to promote tumor growth, early metastasis and chemo-radiation resistance [9,14,15,16]. In PDAC, desmoplasia is also an important niche for the cancer stem cells (CSCs), which drive tumor heterogeneity and influence tumorigenesis, metastasis and drug resistance through their capabilities for self-renewal and multi-lineage differentiation (stemness) (for review see [17]).

Recent studies have demonstrated that the stromal ECM composition “per se” produces important cues that guide the expression of different PDAC phenotypes in both parenchymal cancer cells (CPCs) [5,18,19] and CSCs [5,19]. In particular, ECM composition differently regulates growth, morphology, invasive, angiogenic capacities and secretome profiles in PDAC CPCs and their derived CSCs [5]. Importantly, in that study, only the CSCs secreted factors known to activate and maintain CAFs [19,20,21]. This suggests that the described dual "symbiotic", mutual support interaction between tumor cells and CAFs is maintained primarily through the CSC population through the existence of a tighter relationship between CSCs and CAFs than between the CPCs and CAFs. However, this has yet to be demonstrated.

Once activated, CAFs enhance the development, progression and invasion of PDAC through their extensive crosstalk with the tumor, resulting in reciprocal stimulation and therapy resistance [22,23]. Recent data in PDAC has shown that CAF cells, via their secretome, can increase parenchymal tumor cell (CPC) invasion [24,25,26,27], reduce their growth [24,28] and modify their epigenetic and metabolic phenotypes [25,28]. Only one study [28] measured the effect of the combination of CAF Conditioned Medium (CM) with high levels of ECM collagen I on parenchymal PDAC cell lines (CPCs) but did not determine the individual roles of collagen I and the CAF Conditioned Medium. Importantly, those in vitro experiments were not performed on CSCs and, therefore, the effect of CAFs on CSC behavior in PDAC is still unknown. Nor is the contribution of the ECM in the modulation of the CAF-driven determination of CPC and CSC phenotypic plasticity and behavior known.

Given the complex interactions between cancer cells and CAFs, more work is needed to investigate the contributions of CAFs in enabling or maintaining hallmark behaviors in CSCs, such as growth, invasive capacity and vasculogenic capacity. Here, we analyzed the role of different ECM compositions in modulating the effect of CAF-derived signals from conditioned medium of primary cultured CAFs derived from patients with PDAC on the parenchymal (CPC) and CSC populations. These experiments were performed in a previously described three-dimensional (3D) organotypic model of PDAC [5,18,19] mimicking the ECM of early (low collagen I levels) and late (high collagen I levels) stage PDAC tumors. 

These data reveal that the cellular (CAFs) and acellular (ECM) stromal components interact to differently modulate the hallmark tumor behaviors in the CSCs and CPCs. In particular, the CAFs reduce the growth of CPCs while favoring the growth of CSCs, which would trigger a positive feedback mechanism to stimulate CSC growth and make for a more malignant, persistent and immortal tumor.

## 2. Materials and Methods

### 2.1. Cell Lines

The Panc1 and MiaPaca2 human PDAC parenchymal cell (CPC) lines and their CSCs, generated as previously described [29], were grown and maintained in standard conditions as previously described [5,19]. Panc1 and MiaPaca2 cells are mutated differently in the PDAC driver genes KRAS, CDKN2A, MAP2K4, and TP53 [30] and both cell lines are well established and widely used models in PDAC research [31].

### 2.2. Cancer Associated Fibroblasts (CAFs)

CAFs isolation and culture were processed as previously described [32]. Briefly, pancreatic tissues were obtained during pancreatic surgery from patients with resectable pancreatic adenocarcinoma. The experimental procedure relating to the use of patient-derived pancreatic tumor pieces was performed after approval from the South Mediterranean Personal Protection Committee, under the reference 2011-A01439-32. The tumors were cut into small pieces of 1 mm^3^ using a razor blade. The tissue pieces were dissociated using the Tumor Dissociation Kit (Miltenyi Biotec, Bergisch Gladbach, Germany; 130-095-929) according to the manufacturer’s recommendations. Cells were then suspended, passed through a cell strainer (100 μM) and, finally, plated into a T75 flask. Tissue blocks trapped in the cell strainer were seeded in 10 cm^2^ culture dishes to isolate more CAFs by outgrowth. Cells were cultured in DMEM/F12 medium (Invitrogen, Waltham, MA, USA; 31330-038), 10% serum (Sigma-Aldrich, St. Louis, MI, USA; F7524), 2 mmol/l l-glutamine (Invitrogen; 25030-024), 1% antibiotic-antimycotic (Invitrogen; 15240-062) and 0.5% sodium pyruvate (Invitrogen; 11360-039), and used between passages 4 and 8. Primary CAF were verified by positive α-SMA staining and negative KRT19 immunofluorescence staining. 

For the collection of conditioned media (CM), 1.5 × 10^5^ CAF cells/well were seeded in 24-well cell culture plates. Medium was changed every 3 days. When the monolayer reached approximately 80% confluence, they were incubated with 1 mL of medium with 1% FBS, and without growth factors or antibiotics for 30 h. The conditioned media (CM) were collected, centrifuged and the protein concentration was measured for each with the Bradford protein assay reagent (Pierce, Milan, Italy) using bovine serum albumin as a standard and stored in liquid nitrogen.

### 2.3. D Culture Models

The 90% Matrigel-10% Collagen I and 20% Matrigel-80% Collagen I ECM mixtures were prepared as previously described [5,19]. In all cases, 100 µL of the mixture was plated in 96-well cell culture plates, which was then incubated at 37 °C with 5% CO_2_ for 1 h to allow the mixture to create a thin gel on the bottom of the wells. 1.5 × 10^4^ cells/well were seeded on the top of the matrix and cultured as described above.

### 2.4. Indirect Co-Culture

To condition cells, both CPCs and CSCs were grown for 1 day on 90%M:10%C or 20%M:80%C in their corresponding complete culture media and for the subsequent 5 days in either 100% CM collected from the primary patient CAFs or in the CM diluted at 50% with the same complete culture media used to grow the cells. A change of medium was conducted midweek. To analyze if the CAF CMs could change the growth and/or the growth phenotype (morphology) of the cells, growth was assessed by the Resazurin cell viability assay and morphology were examined microscopically.

### 2.5. Cell Viability Measurements

Cell viability of CPCs and CSCs cultured on the different ECMs were calculated from Resazurin (Immunological Sciences, Rome, Italy) reduction assays as previously described [33] where 10 µL stock Resazurin was added to 100 µL medium and fluorescence was measured after 3 h. The results are normalized to the control as 100 due to variability from experiment to experiment (n = 5) in absolute values of the assay.

### 2.6. Invadopodia Proteolytic Activity

Invadopodia focal ECM proteolysis experiments were conducted in cells seeded onto a layer of either ECM in which quenched BODIPY linked to BSA (DQ-Green-BSA, Thermo Fisher Scientific) was mixed at a final concentration of 30 µg/mL as previously described [5]. Focal proteolysis produces fluorescence in a black background which is used both to quantify proteolytic activity levels and in co-localization analysis. We obtain this by measuring the level (pixel density) of the release of fluorescence underneath the entire cell while the level of focal digestion/proteolysis is obtained by selecting each focal proteolytic point in each cell and measuring the pixel density. The quantity of invadopodia activity was determined with the following measurements: (i) percent of cells with active invadopodia, (ii) number of invadopodia per active cell and (iii) pixel density of digestion performed by individual invadopodia. Mean total actual invadopodia proteolytic activity for 100 cells was then calculated: Invadopodia Proteolysis Index = percentage of Invadopodia-positive cells (proteolitically-active areas also positive for both actin/cortactin) × mean number of invadopodia/cell.

### 2.7. Vascular Network Analysis

CSCs and CPCs were grown on the of 90% Matrigel:10% Collagen I (90%M:10%C) ECM mixture. To study paracrine CAF regulation of vascular parameters, CSCs or parenchymal cells were cultured as above and after 24 h, to permit their adherence, the cultures were incubated with CAF CM as described above for indirect co-culture. After 5 days in these growth conditions, vascular channel networks were photographed using the TE200 microscope (Nikon USA, Garden City, NY, USA) and the development of capillary-like structures (VM) was analyzed as previously described [5].

### 2.8. Statistical Analysis

A two-tailed Student’s *t* test was performed by GraphPad Prism 5 (GraphPad Software, San Diego, CA, USA) assuming unequal variances to compare the effects of CAF CM on Panc1 CPCs and CSCs and to determine whether the differences between two groups were statistically significant. *p*-values < 0.05, 0.01, or 0.001 are indicated as *, ** or ***, respectively when compared to each control for each matrix and ^†^, ^††^ or ^†††^ compared to the same CM treatment on 90% Matrigel:10% Collagen I.

## 3. Results

Given the above-described crosstalk between CAFs and cancer cells and the reported role of the ECM composition in regulating the phenotypes of both PDAC CPCs [5,18,19] and CSCs [5,19], we evaluated the role of the ECM composition on the effect of two concentrations of Conditioned Medium (CM) derived from CAFs isolated twice from two different PDAC patients on the growth, invasive capacity and vascular morphology of each cell type. The experiments were performed with the cells cultured on either 90% Matrigel:10% Collagen I or 20% Matrigel:80% Collagen I since these combinations very well mimic the ECM of early and late stages of PDAC progression, respectively (see Materials and Methods and protocol scheme in Supplemental Figure S1).

### 3.1. CAF Conditioned Medium (CM) Reduces CPC Growth and Increases CSC Growth on All of the Substrates

**Figure 1:**
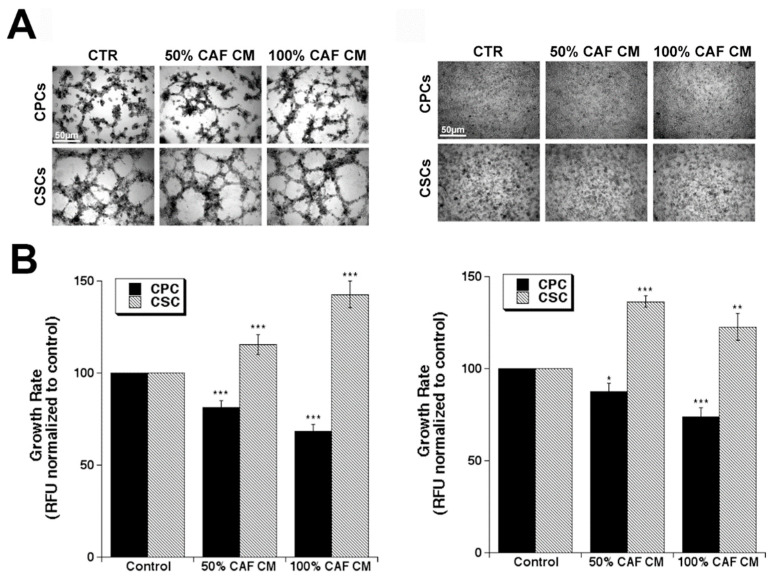
CAF conditioned medium inhibits CPC viability and stimulates CSC viability on both ECM compositions. (**A**) Representative microscopic images of growth morphology of Panc1 CPCs and their derived CSCs after 5 days of culture on organotypic cultures composed of 90% Matrigel:10% Collagen I (left panel) and 20% Matrigel: 80% Collagen I (right panel). Scale bar represents 50 µm for all images. (**B**) CPC and CSC viability in organotypic cultures of 90% Matrigel:10% Collagen I (left panel) and 20% Matrigel:80% Collagen I (right panel) were calculated from Resazurin reduction assays and are normalized to the control as 100 as described in Materials and Methods. Data are mean ± SEM, n = 5, * *p* < 0.05, ** *p* < 0.01, *** *p* < 0.001 to the control of each cell type on each matrix.

When CPCs or CSCs were incubated with two concentrations of CAF CM on the two substrates, we observed that CAF CM reduces growth of the CPCs and increases growth of the CSC in a progressive, dose-dependent manner that was independent of ECM composition with a similar behavior on both early (90%M:10%C) and late (20%M:80%C) ECM compositions (Figure 1) and this same pattern of effect of the patient CAF CM was verified in the MiaPaCa2 cell line (Supplemental Figure S2). This would favor the expansion of the CSCs over the CPCs, in line with the report that only the CSC population secreted factors known to activate CAFs [5] and supports the hypothesis of a mutual “symbiotic” support between these two cell types as in other tumor types [34,35]. Further, this would support the reports in various other tumor types that CAFs drive an increase in general ‘stemness’ of the tumor and would suggest that, at least in PDAC, this occurs by a net favoring of CSC growth over CPC growth.

### 3.2. Effect of Primary CAF Conditioned Medium (CM) on CPC and CSC Invadopodia Proteolytic Activity

**Figure 2:**
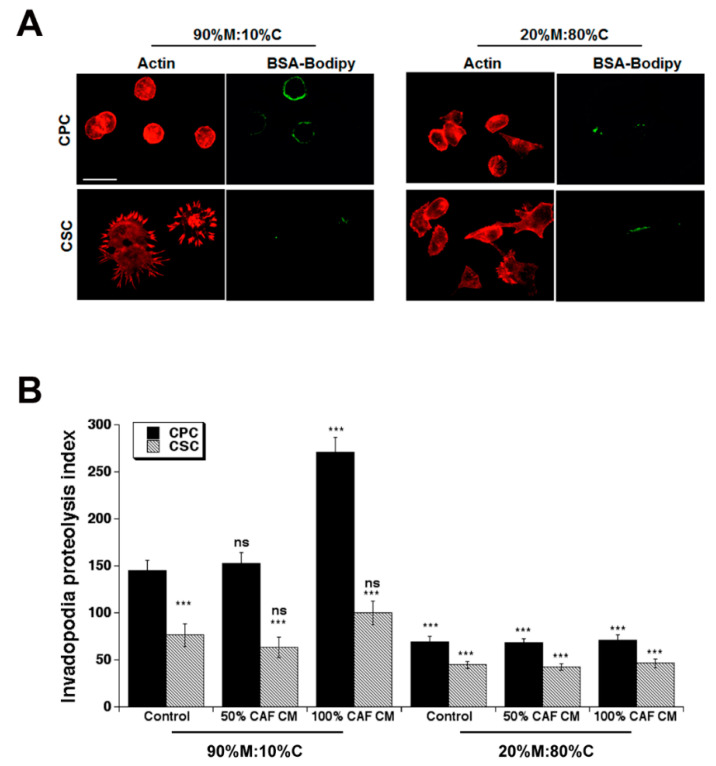
ECM composition modifies the effect of CAF conditioned medium on parenchymal (CPC) and CSC invadopodia ECM degradation. (**A**) Representative microscopic images of actin (red) and invadopodia-dependent proteolysis of BSA-Bodipy dissolved in the ECM (green) as described in the Methods. Scale bar represents 50 µm for all images. (**B**) CPC and CSC invadopodial proteolysis rates in organotypic cultures of 90% Matrigel:10% Collagen I and 20% Matrigel: 80% Collagen I calculated as described in Methods. Data are mean ± SEM, n = 5, ns non significant, *** *p* < 0.001 compared to the control for each cell line on 90% Matrigel:10% collagen I.

We have previously observed that the PDAC CPCs have higher levels of invadopodia-driven invasion than do the CSCs and that this phenomenon is greater on Matrigel rich ECMs than on collagen I ECMs [5]. We, therefore, next analyzed the role of the ECM composition in modulating the CAF CM-dependent regulation of the invasive phenotype of the CPCs and CSCs, by measuring their invadopodia-mediated ECM proteolytic activity (typical experiment in Figure 2A) defined by their Invadopodia Proteolysis Index (Figure 2B) as described in the Materials and Methods [36]. Firstly, as previously reported [5], we found that (i) the control CPCs had a much higher ability to form functional invadopodia and digest the two types of ECM compared to control CSCs and (ii) both cell types had a higher invadopodia proteolytic index when cultured on 90%M:10%C with respect to 20%M:80%C.

The effects of the CAF CM on the formation and activity of invadopodia were very complex. On 90%M:10%C, treatment with 50% CM produced a small but not significant increase in the CPC invadopodia activity while 100% CM stimulated CPC invadopodial activity 2.5-fold. CAF CM had no significant effect on CSC invadopodia activity at either concentration. Interestingly, when cultured on 20%M:80%C neither cell line responded significantly to CAF CM at either concentration. These dynamic trends of CAF CM on invadopodia activity were confirmed in the MiaPaC2 cell line on 90%M:10%C (Supplementary Figure S3).

### 3.3. Effect of Primary CAF Conditioned Medium (CM) on CPC and CSC Vasculogenic Mimicry

We have previously reported that vascular-like structures correspondent with in vivo Vasculogenic Mimicry (VM) were formed by the CSCs on 100% Matrigel and were reduced as Collagen I levels increased and completely disrupted as Collagen I levels surpassed 30% of the ECM composition [5]. We asked whether the CAF CM-driven stimulation of CSC growth observed in Figure 1 would also result in an increase of VM organization in the CSCs and, perhaps, in a slight increase in VM in the CPC population. Indeed, when CSCs or CPCs were incubated with the two concentrations of CAF CM on the 90%M:10%C substrate, we observed that CAF CM increases both the mean number of lacunae per well and number of capillary connections to nodes per field of the CSCs in a progressive, dose-dependent manner that resulted in the formation of very tight, organized vascular structures. Interestingly, CAF-CM somewhat stimulated a capillary-like phenotype also in the CPCs but only at the higher concentration of CAF CM, suggesting that it may induce the epithelial-mesenchymal transition of CPCs. Again, these results were verified in the MiaPaca2 CSC cell line (Supplementary Figure S4).

## 4. Discussion

Human PDAC is characterized by desmoplasia, an extensive Collagen I-rich and fibrotic ECM within which are embedded heterogeneous cell populations of both cancer parenchymal (CPCs) and cancer stem (CSCs) cells and the various accessory cell types of which the CAFs are of particular importance. During PDAC progression, the normal laminin-rich basal membrane is disrupted and desmoplastic fibrosis becomes more abundant as the tumor progresses [37] and the direct exposure to the increased interstitial collagen I drives enhanced metastasis and poor prognosis [14,38].

However, tumor growth rate and phenotypes are influenced not only directly via their own intrinsic (gene expression-related) and extrinsic (ECM-related) factors, but also via the interaction of the tumor cells with the accessory cells found in the tumor stroma. In PDAC, the major stroma cell type driving progression is considered to be the Cancer Associated Fibroblasts (CAFs) and due to the sparse distribution of tumor cells the interaction between the different cell types occurs principally via the soluble factors secreted by both tumor cells and CAFs [24,25,26,27]. While in other tumor types, the interaction of CSCs with CAFs has been documented, in PDAC the role of CAFs in determining and/or modulating the balance between the parenchymal and CSC tumor populations is still unknown.

**Figure 3:**
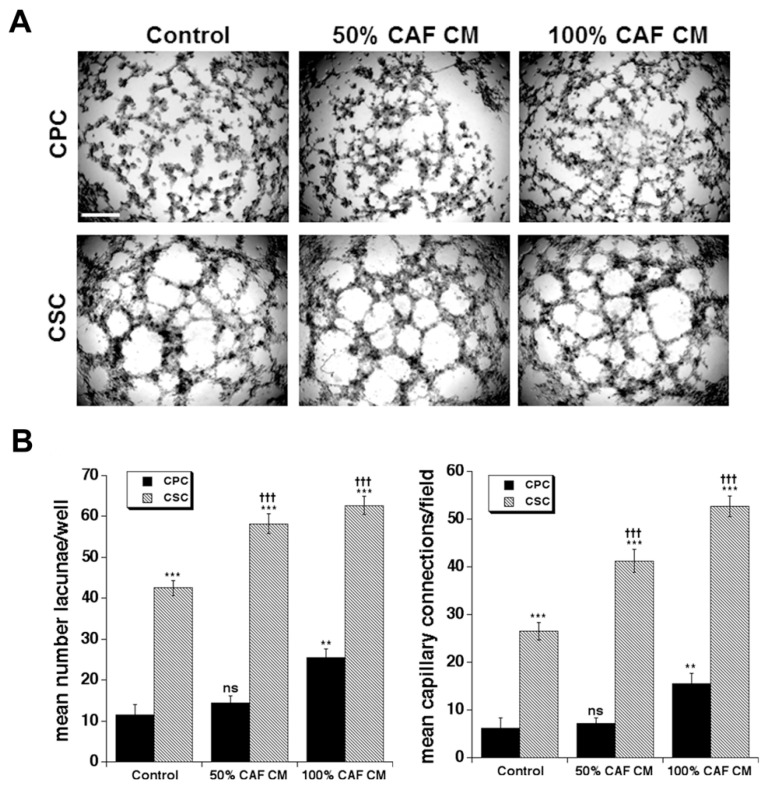
CAF conditioned medium stimulates the vascular-like morphology (VM) of both CSCs and CPCs grown on 90% Matrigel:10% Collagen I. ECM composition modifies the effect of CAF CM on vasculogenic mimicry in CPCs and CSCs. Cells were grown on 90% Matrigel: 10% Collagen I and after 24 h to permit their adherence the cultures were incubated with CAF conditioned medium and the cells cultured an additional 5 days and VM measured as described in Materials and Methods. (**A**) Representative microscopic images of growth morphology of CPCs and their derived CSCs cultured on organotypic cultures composed of 90% Matrigel:10% Collagen I for 5 days with their growth medium or with either 50% or 100% of the CAF conditioned medium. Scale bar represents 50 µm for all images. (**B**) After 6 days in these growth conditions, vascular channel networks were analyzed as described in Material and Methods for mean number of lacunae per well (left panel) and mean number of capillary connections per field (right panel). Mean ± SEM from three independent experiments, ns, non significant, ** *p* < 0.01, *** *p* < 0.001 compared to the CPC control; ^†††^ *p* < 0.001 CSCs compared to their control.

Here, utilizing organotypic cultures mimicking the changes in ECM composition with PDAC progression, we characterized the role of CAFs on CPC and CSC phenotypes on ECM compositions known to drive specific growth and phenotype patterns in the two tumor cell types. Importantly, we utilized conditioned medium from primary CAFs isolated from PDAC patient’s tumors to better reflect the complexity of the stromal tumor microenvironment. We find that when growing on an early tumor ECM (modeled by 90%M:10%C), the CAFs increased both CSC growth (Figure 1A) and their assembly into a VM network (Figure 3). Together, this increases their dedicated programing towards the preparation of a vascular niche and eventual transdifferentiation into an endothelial-like network [5]. At the same time, when on Matrigel the CAFs further reduced the growth rate of the more differentiated CPC cell population (Figure 1B), but greatly increased their already high invadopodia-driven invasive capacity (Figure 2). This concerted over-activation of these two malignant phenotypes by the CAFs, i.e., (1) the high rate of CPC local invasion into the (2) CSC-derived vasculogenic network suggest that the CAFs specifically activate the previously described symbiotic relationship between the parenchymal CPC cells and the CSCs that underlies early PDAC infiltration and metastasis [5].

These data further support the idea that CAFs contribute to drive progression by further activating the parenchymal tumor cell (CPC) and CSC behaviors that underlie another important PDAC characteristic, the very early development of metastasis even before the primary tumor can be detected [39]. Indeed, it has been reported [5] that growth on Matrigel-rich ECM activates the CSC program driving their transdifferentiation into an endothelial-like VM network via a VEGF/VEGFR-2 mediated cascade while the more differentiated CPCs have a high invadopodia-driven invasive capacity that is stimulated by EGF [40], which is highly secreted by the CSCs. In this way, parenchymal CPC cells and CSCs interact to contribute to the vascular [41] and invasive phenotype of the early-stage tumor.

Here we find that the CAFs further activate these two malignant phenotypes to exacerbate these parenchymal CPC and CSCs cell interactions that contribute to the vascular [41] and invasive phenotype of the early stage tumor. Based on the findings reported here, this concerted and reciprocal activation of these two malignant phenotypes is further stimulated by a third factor, the secretion by CAFs of various pro-angiogenic and growth factors. Indeed, CAFs secrete many factors, including TGF-ß1, PDGF, FGF-2, various interleukins, CXCL8 and VEGFA [24,25,42] and CXCL1 [33]. Altogether, these factors support high CSC growth rate necessary to form the vascular network and high CPC invasiveness. This would both increase symbiotic relationship between the CPCs and the CSCs in which the high rate of local invasion executed by CPCs into the aberrant vascular network created by the CSC-derived vascular system (together with endothelial cells, pericytes, etc.) [42] that underlies the initiation and maintenance of early PDAC infiltration and metastasis. 

This enhanced ability of the CAFs to stimulate the previously described CSC growth/pro-angiogenic program [5], especially in response to an early stage tumor microenvironment (e.g., growth on Matrigel), is in line with in vivo experiments in which (a) subcutaneous tumors originating from CSCs gave rise to a more abundant vascular network composed of larger vessels than the tumors originating from CPCs, (b) the tumors of the CSC-injected mice had increased Ki-67 staining for mitotic index [5] and (c) grow faster than those in mice injected with CPCs [29].

## 5. Conclusions

**Figure 4:**
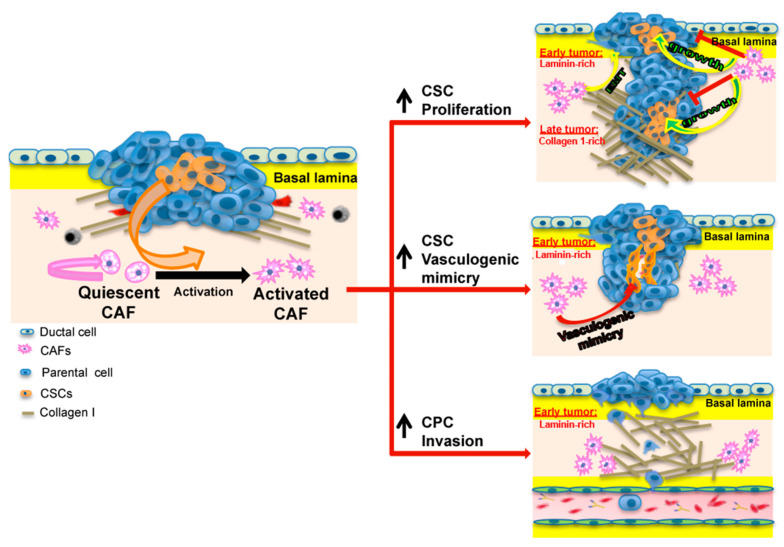
Model of Influence of the ECM composition on the CAF-dependent regulation of CPC and CSC plasticity. Matrigel induces the formation of autocrine loops in CSCs. Indeed, on Matrigel CSCs secrete a high amount of potent proangiogenic and growth factors (i.e., PDGF, MMP9, IL8, EGF, HGF, bFGF, ET-1) [5], which, via paracrine mechanisms, stimulate CAF growth [20], which in turn secrete factors that increase CSC growth/self-renewal and vasculogenic mimicry while decreasing CPC growth [24,28] but increasing CPC invasion [24,25,26,27]. This creates a vicious positive-feedback growth cycle between the CAFs and CSCs to increase the stemness of the tumor while exacerbating the aggressive angiogenesis phenotype of the CSCs and invasive phenotype of the CPCs.

In conclusion, this study sheds further light on the role of the CAFs in driving the very early metastatic development, one of the most representative PDAC hallmarks [39]. We find that the CAFs and acellular stromal components interact to modulate the hallmark tumor behaviors of the CPC and CSC cell types and drive metastatic progression by stimulating the hallmarks of each tumor cell type that contribute to metastasis [5,6,7,9,11,14]: invasion in the CPCs and growth & angiogenesis in the CSCs (Figure 4). Altogether, these findings propose a scenario in which the ECM composition and the cellular secretome of the CAFs cooperate to jointly regulate both growth and morphology of the CPC and CSC cell lines and, by modulating the highly dynamic interactions between them, establishes a continuum between tumor initiation and progression in primary PDAC tumors. We suggest future studies using an ex in vivo model in-order-to better understand internal variability of both cancer cells and CAFs, and their interactions as the ex in vivo model provides a fully intact tumor microenvironment. 

## Data Availability

Not applicable.

## Supplementary Materials

The following supporting information can be downloaded at: https://www.mdpi.com/article/10.3390/cancers14153737/s1, Figure S1: Scheme for the experimental protocol for indirect co-culture of PDAC tumor CAFs with PDAC parenchymal cells (CPCs) or Cancer Stem Cells (CSCs).; Figure S2: CAF conditioned medium inhibits CPC viability and stimulates CSC viability.; Figure S3: ECM composition modifies the effect of CAF conditioned medium on parenchymal (CPC) and CSC invadopodia ECM degradation.; Figure S4: CAF conditioned medium stimulates the vascular-like morphology (VM) of both CSCs and CPCs grown on 90% Matrigel:10%Collagen I. ECM composition modifies the effect of CAF CM on vasculogenic mimicry in CPCs and CSCs.
