# Supplementary material for: Cancer Associated Fibroblast (CAF) Regulation of PDAC Parenchymal (CPC) and CSC Phenotypes Is Modulated by ECM Composition
Source: Cancers (Basel). 2022 Jul 31;14(15):3737. doi: 10.3390/cancers14153737 (PMC9367491; doi:10.3390/cancers14153737)
Supplement: Supplementary file 1 [file cancers-14-03737-s001.zip › cancers-1812567-supplementary.pdf]

cancers

Supplementary Materials

Cancer Associated Fibroblast (CAF) Regulation of PDAC Parenchymal (CPC) and CSC Phenotypes Is Modulated by ECM Composition

Stefania Cannone, Maria Raffaella Greco, Tiago M. A. Carvalho, Helene Guizouarn, Olivier Soriani, Daria Di Molfetta, Richard Tomasini, Katrine Zeeberg, Stephan Joel Reshkin and Rosa Angela Cardone

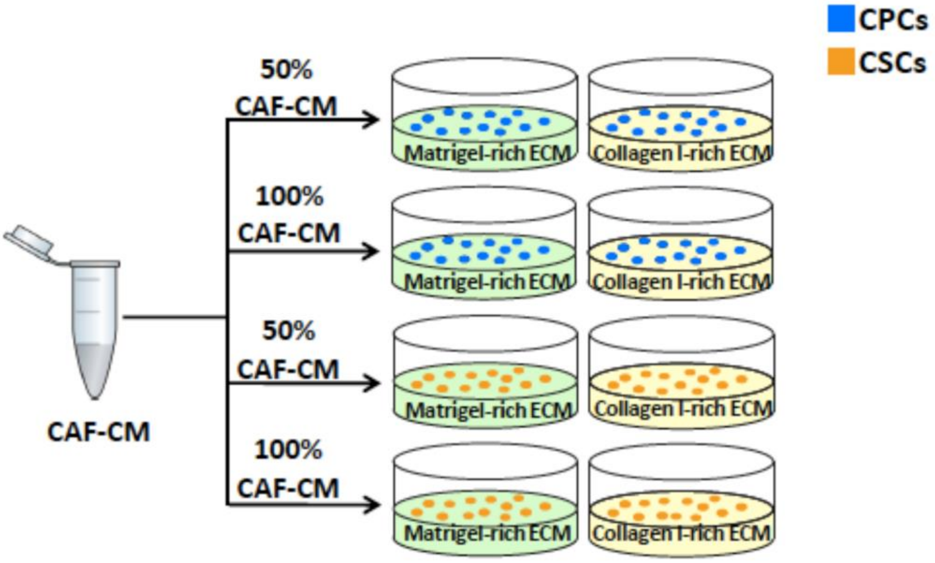

Figure S1. Scheme for the experimental protocol for indirect co-culture of PDAC tumor CAFs with PDAC parenchymal cells (CPCs) or Cancer Stem Cells (CSCs).

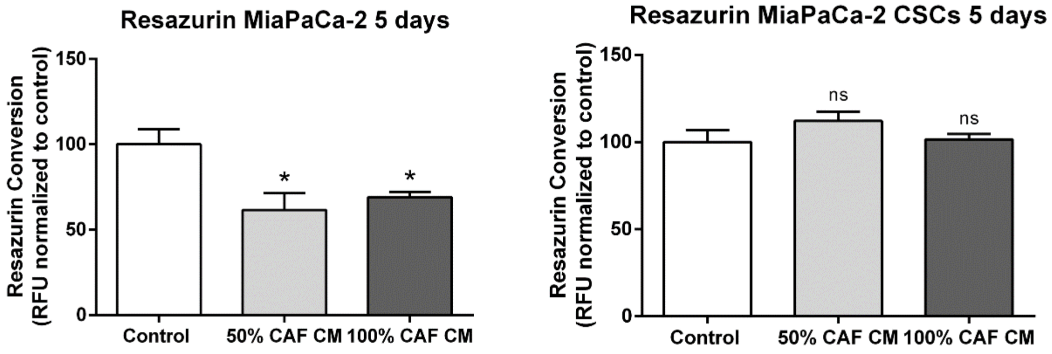

Figure S2. CAF conditioned medium inhibits CPC viability and stimulates CSC viability.

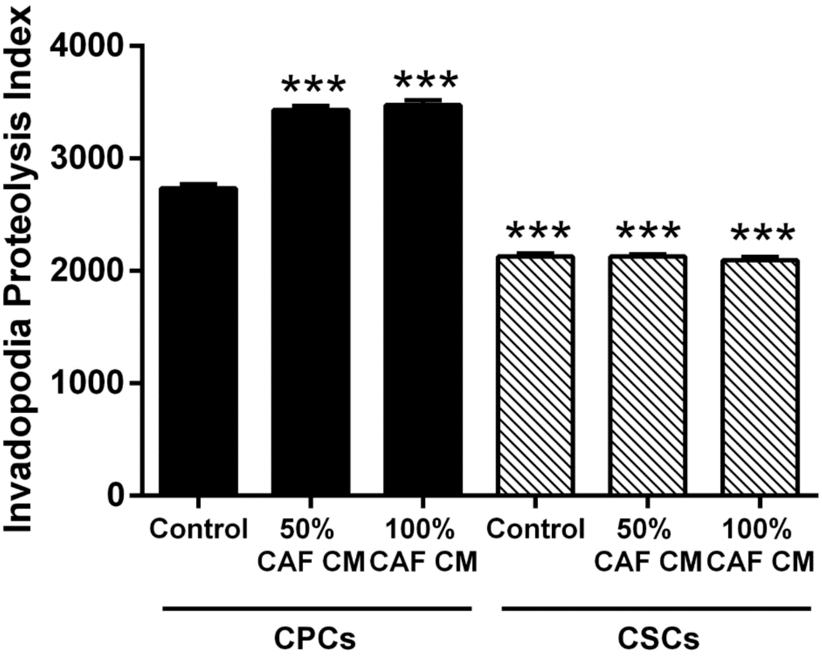

Figure S3. ECM composition modifies the effect of CAF conditioned medium on parenchymal (CPC) and CSC invadopodia ECM degradation.

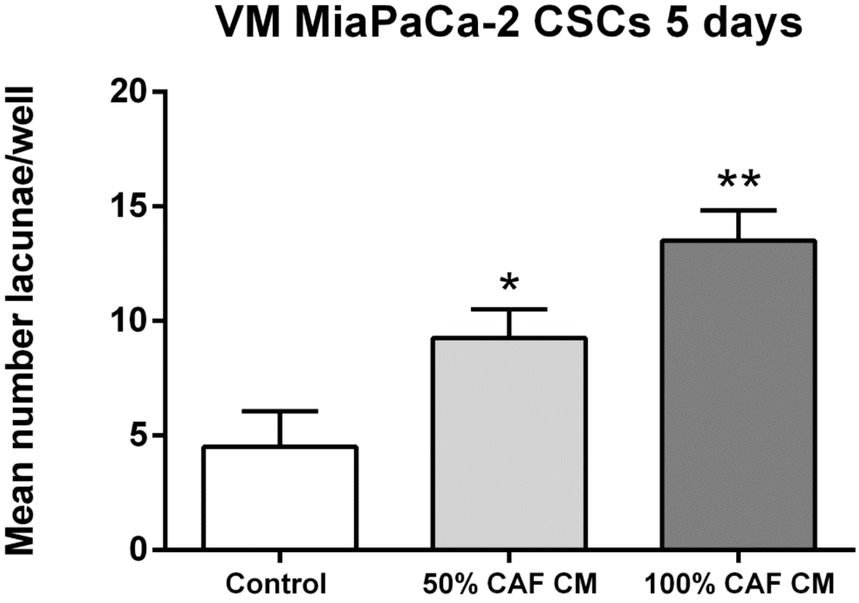

Figure S4. CAF conditioned medium stimulates the vascular-like morphology (VM) of both CSCs and CPCs grown on 90% Matrigel:10%Collagen I. ECM composition modifies the effect of CAF CM on vasculogenic mimicry in CPCs and CSCs.
